# Hydrogen Sulfide-Mediated Polyamines and Sugar Changes Are Involved in Hydrogen Sulfide-Induced Drought Tolerance in *Spinacia oleracea* Seedlings

**DOI:** 10.3389/fpls.2016.01173

**Published:** 2016-08-04

**Authors:** Juan Chen, Yu-Ting Shang, Wen-Hua Wang, Xi-Yan Chen, En-Ming He, Hai-Lei Zheng, Zhouping Shangguan

**Affiliations:** ^1^State Key Laboratory of Soil Erosion and Dryland Farming on the Loess Plateau, Northwest A&F UniversityYangling, China; ^2^Fujian Key Laboratory of Subtropical Plant Physiology and Biochemistry, Fujian Institute of Subtropical BotanyXiamen, China; ^3^College of Life Science, Northwest A&F UniversityYangling, China; ^4^Key Laboratory of the Ministry of Education for Coastal and Wetland Ecosystem, College of the Environment and Ecology, Xiamen UniversityXiamen, China

**Keywords:** hydrogen sulfide, drought, soluble sugar, polyamines, water relations, *Spinacia oleracea*

## Abstract

Hydrogen sulfide (H_2_S) is a newly appreciated participant in physiological and biochemical regulation in plants. However, whether H_2_S is involved in the regulation of plant responses to drought stress remains unclear. Here, the role of H_2_S in the regulation of drought stress response in *Spinacia oleracea* seedlings is reported. First, drought stress dramatically decreased the relative water content (RWC) of leaves, photosynthesis, and the efficiency of PSII. Moreover, drought caused the accumulation of ROS and increased the MDA content. However, the application of NaHS counteracted the drought-induced changes in these parameters. Second, NaHS application increased the water and osmotic potential of leaves. Additionally, osmoprotectants such as proline and glycinebetaine (GB) content were altered by NaHS application under drought conditions, suggesting that osmoprotectant contributes to H_2_S-induced drought resistance. Third, the levels of soluble sugars and polyamines (PAs) were increased differentially by NaHS application in *S. oleracea* seedlings. Moreover, several genes related to PA and soluble sugar biosynthesis, as well as betaine aldehyde dehydrogenase (*SoBADH*), choline monooxygenase (*SoCMO*), and aquaporin (*SoPIP1;2*), were up-regulated by H_2_S under drought stress. These results suggest that H_2_S contributes to drought tolerance in *S. oleracea* through its effect on the biosynthesis of PAs and soluble sugars. Additionally, GB and trehalose also play key roles in enhancing *S. oleracea* drought resistance.

## Introduction

Drought induces water scarcity and osmotic stresses that restrict the growth and development of plants because water absorption by plant roots from the soil is inadequate to satisfy plant transpiration demand (Blum, 1996). Additionally, leaf cell turgor is significantly lessened by water deficiency, which in turn restricts cell expansion, the increase of leaf area, and photosynthesis, thus inhibiting the accumulation of biomass (Chaves et al., 2003). An effective strategy for plant drought adaptability has been to inhibit water scarcity caused by transpiration, which saves soil moisture and allows plants to sustain sufficient water content to maintain key physiological processes (Yoo et al., 2010).

During water deficiency and other osmotic stresses, the accumulation of compatible low-molecular-weight osmoprotectants, such as soluble sugars (including glucose, fructose, and sucrose), proline, sugar alcohols, special amino acids and glycinebetaine (GB) may confer stress tolerance to plants (García-Mata and Lamattina, 2001; Rivero et al., 2014). Additionally, the composition and content of the osmoprotectants in stressed plants can vary considerably depending on the species and environmental conditions (Rivero et al., 2014). Moreover, polyamines (PAs), including putrescine (Put), spermidine (Spd) and spermine (Spm), are ubiquitous biological organic-amines involved in plant growth, development and the response to biotic and abiotic stresses (Takahashi and Kakehi, 2010). When plants are exposed to stress conditions such as drought, heat, chilling, or salinity, PA levels significantly increase and become one of the most prominent metabolic features (Takahashi and Kakehi, 2010; Tiburcio et al., 2014). These changes are caused mainly by the biosynthesis of PAs, PA oxidation, and/or interactions with other pathways under stress conditions (Tiburcio et al., 2014). The PA metabolic pathway mediates nitrogen or carbon metabolism and interacts with other metabolites, such as stress-protective compounds including signaling molecules and hormones (Moschou et al., 2012; Tiburcio et al., 2014). As an example, the abscisic acid (ABA)-induced accumulation of PAs enhances PA oxidation, which in turn enacts protection against such stresses as against drought in plants (Toumi et al., 2010). Additionally, many previous studies have shown that PAs play an important role in salt tolerance and drought resistance in plants (Alcázar et al., 2010b; Yin et al., 2013, 2015). Under drought stress, plants with high PA contents exhibit strong adaptability, and exogenous PAs alleviate drought stress. All these results demonstrate that PA levels are positively correlated to drought resistance in plants (Capell et al., 2004). Recently, much research has indicated that the overexpression of PA biosynthesis-related genes increases PA content and improves drought tolerance or salt stress (Capell et al., 2004; Wen et al., 2008; Alcázar et al., 2010a). Moreover, knockout/knock-down mutants whose capacity to synthesize PAs were abolished or limited were more sensitive to drought or salt stress than wild-type plants (Kasinathan and Wingler, 2004). Together, these studies indicate that PAs play a vital role in regulating drought tolerance and salt resistance in plants.

The small bioactive gaseous hydrogen sulfide (H_2_S) exhibits both physical and functional similarities with other gasotransmitters such as carbon monoxide (CO) and nitric oxide (NO) and has been shown to be involved in different physiological processes in animals (Hosoki et al., 1997; Zhao et al., 2001; Wang, 2002; Li et al., 2006; Yang et al., 2008). NaHS, a donor of H_2_S, has been used to study the physiological function of H_2_S in animals and plants (Hosoki et al., 1997; Zhang et al., 2008; García-Mata and Lamattina, 2010). Recently, much of this research has focused on the physiological function of H_2_S in plants. Specifically, H_2_S promotes seed germination, protects against copper-induced oxidative damage, counteracts chlorophyll loss, and alleviates oxidative damage from water scarcity in sweet potato leaves (Zhang et al., 2008, 2009). Additionally, boron, salinity and aluminum toxicities were alleviated by H_2_S treatment (Wang et al., 2010, 2012; Zhang et al., 2010b; Chen et al., 2013, 2015a). Furthermore, low concentrations of H_2_S regulated flower senescence and promoted the embryonic root length of *Pisum sativum* (Li et al., 2010; Zhang et al., 2011). In addition, García-Mata and Lamattina (2010) reported that H_2_S regulated the ATP-binding cassette (ABC) transporter in guard cells and then caused stomatal closure; these processes are closely connected to the ABA signaling pathway. Our previous study also showed that H_2_S promoted iron availability in *Zea mays* seedlings and enhanced photosynthesis in *S. oleracea* seedlings (Chen et al., 2011, 2015b). Moreover, many previous studies have shown that H_2_S can effectively improve drought tolerance in many different plants (Zhang et al., 2010a; Shan et al., 2011; Jin et al., 2013). For example, Ziogas et al. (2015) reported that H_2_S pre-treatment led to the post-translational modification (PTM) of proteins under drought stress in citrus plants. Additionally, Li et al. (2015) showed that H_2_S alleviated drought-induced PSII damage due to fast D1 protein turnover. A previous study also showed that H_2_S regulated the expression of drought-related genes including *DREB* and *RD29A* (Jin et al., 2011). The aforementioned studies focused on only the protection against drought-induced oxidative damage H_2_S offered via the enhancement of non-enzymatic and enzymatic antioxidant capacities, the reduction of evaporation, or the control of stomatal conductance in plants. However, the detailed mechanisms of the effects of H_2_S on drought stress remain unclear, especially with respect to physiological processes in plants.

Because PAs and soluble sugars particularly enhance drought tolerance, the question of whether PAs and soluble sugars are involved in H_2_S-induced drought tolerance needs to be addressed. Recent studies have shown that the bioactive free radical molecule NO and PAs share certain overlapping physiological roles in plants (Yamasaki and Cohen, 2006). The synthesis of NO was induced by PAs in *Arabidopsis*, which indicates that NO might contribute to PA signaling during stress (Tun et al., 2006). However, whether a low concentration of H_2_S similar to that of NO has an effect on PA levels and further enhances the resistance of plants to stress remains unclear.

In this study, we present important data that reveals a novel effect of H_2_S in plant physiology, more specifically on drought stress. These results support the suggestion that H_2_S significantly enhances the tolerance of plants to drought stress through its effects on PA and soluble sugar contents, which change the expression levels of genes associated with PAs and sugar biosynthesis.

## Materials and Methods

### Plant Culture and Treatment

Seeds of *Spinacia oleracea* were first sterilized by immersion in 75% ethanol for 3 min followed by 10 min in a 10% sodium hypochlorite solution. Next, seeds were washed with distilled water and germinated in a soil/vermiculite (1:1) mixture. Subsequently, 1-week-old seedlings were transferred to plastic pots (15 cm × 15 cm; 25 seedlings per pot) filled with a soil/vermiculite (1:1) mixture The plants were then grown in a controlled growth chamber with a light/dark regime of 10/14 h, a relative humidity of 80%, a temperature of 21/27°C, and a photosynthetically active radiation (PAR) of 190 μmol m^-2^ s^-1^ and were watered with a ½ strength Hoagland solution every other days.

NaHS was used as the exogenous H_2_S donor as described by Hosoki et al. (1997). Six-week-old seedlings were treated with the ½ strength Hoagland solution containing 100 μM NaHS. The same volume ½ strength Hoagland solution was used as the control. For drought treatment, 6-week-old plants subjected to drought stress, were grown in pots by withholding water for 8 days, and then re-watered until the substrate was saturated. Treated leaves were sampled after 0, 5, and 8 days of drought stress and after 1 and 4 days of re-watering. All samples were frozen rapidly in liquid nitrogen and stored at -80°C.

### Measurement of Soil Water Content, Plant Survival, Leaf Relative Water Content, and Detached Leaf Water Loss

Drought stress was imposed by withholding water from containers of soilless media (with dry weight of 58.6 ± 5.4 g) containing 25 plants (6-week-old). The containers were irrigated with water to saturation and weighed at the start of the drought stress treatment (initial weight) and then periodically throughout the treatment period. Relative soil water content (SWC) was calculated as follows: (final fresh weight-dry weight)/(initial weight-dry weight) × 100. After 8 days of drought, the plants were re-watered, and the plant survival was measured 4 days after re-watering (Yoo et al., 2010).

In a separate experiment, the relative water content (RWC) of fully expanded leaves was assessed in 6-week-old plants growing at different relative SWC. Leaves were cut and immediately weighed to obtain the leaf FW. The leaves were then placed into small bottles filled with distilled water for 24 h, blotted up to remove excess water, and then weighed to obtain the leaf turgid weight (TW). The leaves were then dried to a constant weight at 70°C and reweighed to obtain the leaf dry weight (DW). The leaf RWC was calculated as follows: (FW-DW)/(TW-DW) × 100 (Yoo et al., 2010).

The detached leaves were treated with different concentrations of NaHS (0, 10, 100, 500, and 1000 μM) for 3 h and the leaf RWC was measured according to the method of Yoo et al. (2010). Water loss in the detached leaf was determined according to the method of Whittaker (1994). Leaves were excised from plants, weighed to determine their initial weight, placed with their adaxial surfaces upward on a petri dish in a light incubator (at a temperature of 25 ± 2°C and a relative humidity of 60%) and reweighed at 30 min intervals for up to 6 h to obtain the actual time fresh weight. The water loss was calculated as follows: (initial weight-actual time fresh weight)/initial weight × 100.

### Measurement of Endogenous H_2_S Concentration

Endogenous H_2_S was measured by the formation of methylene blue from dimethyl-*p*-phenylenediamine in H_2_SO_4_ according to the method described by Sekiya et al. (1982) and Zhang et al. (2009) with some little modifications. Leaves (0.3 g) were ground and then extracted with 3 ml of phosphate buffer solution (pH 6.8, 50 mM) including 0.2 M ascorbic acid and 0.1 M EDTA. Subsequently, 1 M HCl (0.5 ml) was added to the homogenate in a test tube for releasing H_2_S, and 0.5 ml of 1% (w/v) zinc acetate was used to absorb H_2_S. After 30 min of reaction, 5 mM dimethyl-*p*-phenylenediamine (0.3 ml) dissolved in 3.5 mM H_2_SO_4_ was added to the trap. Then, 50 mM ferric ammonium sulfate in 100 mM H_2_SO_4_ was injected into the trap. The amount of H_2_S in the zinc acetate traps was determined at OD 667 nm after incubating the mixture for 15 min at room temperature. Blanks were prepared using the same procedures without the zinc acetate solution.

### Gas Exchange and Chlorophyll Fluorescence Quenching Analyses

The gas exchange of fully expanded leaves (the number leaves of a plant was about 12–14), including transpiration, stomatal conductance, and net CO_2_ assimilation, was determined using a portable photosynthesis system (Li-6400, Li-Cor, Lincoln, NE, USA). Plants were grown for 6 weeks under short-day conditions (10 h light). Such conditions resulted in plants with leaves large enough to fill the 6-cm^2^ Li-6400 chamber. Gas exchange was measured at PAR levels of 1200, 800, 600, 400, 200, 100, 50, 20, 10, and 0 μmol m^-2^ s^-1^. All measurements were conducted in the morning (9:00–11:30) to avoid the high temperature and air vapor pressure deficits in the afternoon. Light was supplemented using an LED light system. Using non-rectangular hyperbola modeling, the response of leaf Pn to PAR was calculated with respect to the apparent dark respiration (*R*d), light compensation point (*L*cp), light saturation point (*L*sp), apparent quantum yield (*AQE*) and maximal net photosynthetic rate (*P*max), as described by Prioul and Chartier (1977).

$$Pn = AQE\times PAR+P\max-\frac{\sqrt{{\left(AQE\times PAR+P\max\right)}^{2}-4AQE\times \theta \times PAR\times P\max}}{2\theta }-Rd,$$

where *𝜃* is the convexity, quantum efficiency is the initial slope of the curve, and *R*d is the point at which the curve crosses the *y* axis at PAR = 0, and the *L*cp at which the curve crosses the *x* axis. The vapor pressure deficit (VPD) during measurement was ∼1 kPa. The instantaneous water use efficiency (WUE) was calculated as the net CO_2_ assimilation rate divided by the transpiration rate.

Chlorophyll fluorescence is considered a tool for interpreting the stress tolerance of plants by evaluating the physiological status of the plant and the state of Photosystem II (PSII) (Krause and Weis, 1991; Peeva and Cornic, 2009). The chlorophyll fluorescence of *S. oleracea* was measured using a Plant Efficiency Analyzer (Hansatech Instruments Ltd., Norfolk, England). The ratio of variable (Fv) to maximum fluorescence (Fm) was measured in four seedlings per pot. The leaves were dark-adapted for more than 30 min prior to the measurement. The minimum fluorescence (Fo), the Fm, the variable fluorescence (Fv = Fm-Fo) and the ratio of Fv/Fm were recorded for 15 s at a 100% intensity level of the photon flux density (4000 μmol m^-2^ s^-1^). Additionally, the steady-state fluorescence level reached upon continuous illumination (Fs′), the maximal fluorescence level induced by a saturating light pulse at the steady-state (Fm′), and the minimum fluorescence level after a 3 s period of far-red light required to oxidize the plastoquinone pool (Fo′) were measured and used to calculate other parameters as follows: The quantum yield of PSII photochemistry (PSII) was calculated as 1-(Fs′/Fm′). The electronic transport ratio (ETR) was calculated as PAR×PSII×0.85×0.5. Non-photochemical quenching (NPQ) was calculated as (Fm/Fm′)-1. Photochemical quenching (qP) was calculated as (Fm′-Fs)/(Fm′-Fo′) (Krause and Weis, 1991).

### Measurement of Leaf Water Potential and Osmotic Potential

Leaf water potential was measured after plants were subjected to drought for 0, 5 and 8 days and re-watered for 1 and 4 days. The fully expanded leaves were selected and measured between 10:00 and 11:00 A.M. using a pressure chamber (Model 1000, PMS Instrument Co., Corvallis, OR, USA). The osmotic potential was measured using a dew point microvolt meter (Model 5520, Wescor, Logan, UT, USA). Leaf water and osmotic potentials were calculated according to the method of Yin et al. (2013). Each treatment included six replicates.

### Measurement of Superoxide Radical, Hydrogen Peroxide and Lipid Peroxidation

The production of superoxide radical ($O_{2}^{–•}$) was measured using the method of Wang and Luo (1990) with some modifications. Hydrogen peroxide (H_2_O_2_) was measured spectrophotometrically according to Alexieva et al. (2001). The lipid peroxidation level was determined in terms of malondialdehyde (MDA) content via the thiobarbituric acid (TBA) reaction as described by Dhindsa et al. (1981).

### Measurement of Osmolyte Content

Proline content was determined according to the method of Bates et al. (1973). Glycinebetaine content was measured using an HPLC Shimadzu-V analytical procedure according to the method of Bessieres et al. (1999) with some modifications. Frozen leaf samples in liquid nitrogen were homogenized with 4 ml water: chloroform: methanol (3: 5: 12 v/v/v) solution and incubated overnight at 4°C. The upper methanolic phase (1 ml) of the extract was purified using BioRad AG1-X8 ion exchange resin. The ion exchange resin was removed by centrifugation at 5000 *g* for 10 min, and the supernatant was filtered using a 0.22 μm membrane filter before being loaded into the HPLC system. A Nucleogel RP column (RP-S 100-8, 300 × 7.7 mm) proceeded by a guard column was used, and the mobile phase (15 mM KH_2_PO_4_) was delivered by an analytical isocratic pump at a flow rate of 0.7 ml min^-1^ at 70°C. The GB content was measured using a UV detector at 192 nm, and quantification was performed by comparing the peak surface areas.

### Measurement of Glucose, Fructose, Sucrose, and Trehalose Content

Glucose and sucrose were measured according to the method of Zhao et al. (2004) using the Shimadzu sugar analysis system (HPLC, Shimadzu, Kyoto, Japan). Leaves were homogenized in 7 ml 85% (v/v) ethanol, the first 5 ml aliquot was adjusted to 1 mM with respect to lactose and used as an internal standard. The slurry was transferred to a 10 ml tube, and the mortar was washed with an additional 2 ml 80% ethanol, which was added to the tube. The suspensions were heated at 80°C for 30 min and centrifuged at 12000 *g* for 20 min, and then the supernatant was collected. The supernatant was heated at 95 °C for 2 h to remove ethanol, and the final sample (∼500 μl) was frozen at -80°C for 24 h. Subsequently, the sample was lyophilized to dryness, reconstituted in 500 μl distilled, deionized water, and centrifuged at 12000 *g* for 20 min. Then, the supernatant was passed through a membrane filter (pore size 0.22 μm) for analysis. Sugars in the extracts were injected into an eluent of 19 mM NaOH delivered at 1 ml min^-1^ using a Waters 1525 HPLC pump (Waters 1525/VIS 2424 Detector, Breeze^TM^, USA) and separated by anion exchange using an XBridge^TM^ amide C18 column (4.6 × 250 mm, 3.5 μm particle size) with guard column. Each treatment included four replicates.

Fructose and trehalose contents were estimated using fructose and trehalose reagent kits, respectively (Shanghai Solarbio Biocompany, Shanghai, China). The absorbances of fructose and trehalose were measured at 530 and 520 nm, respectively, following the manufacturer’s instructions.

### Measurement of Polyamines Content

Polyamines (PAs) were extracted according to the method of Flores and Galston (1982) and Duan et al. (2008). Briefly, leaves were ground in 1 ml of 5% (v/v) cold perchloric acid and then incubated at 4°C for 1 h. After incubation, the homogenate was centrifuged at 12000 *g* for 30 min at 4°C. To measure the conjugated PAs, the plant extract residues were washed with 5% HClO_4_ and then subjected to hydrolysis in 6 M HCl at 110°C for 16 h. The filtered hydrolysates were allowed to evaporate to dryness, and the residues were dissolved in 5% HClO_4_ for the measurement of conjugated PAs. Subsequently, the supernatant and the suspension residues were benzoylated as follows: A 500 μl volume of supernatant was treated with 2 ml of 2 N NaOH and 10 μl benzoyl chloride, and the mixture was then vortexed for 30 s and incubated for 30 min at 37°C. The reaction was terminated by adding 2 ml saturated NaCl solution. Next, the benzoyl polyamine was extracted with 2 ml cold diethyl. Ultimately, 1 ml ether phase was evaporated to dryness and dissolved in 1 ml of methanol for the determination of endogenous polyamines. Eventually, high performance liquid chromatography (Waters 1525/UVD 2489 Detector, Breeze^TM^, USA) was used to analyze the endogenous PA content. Briefly, 20 μl of benzoyl PAs were injected into the loop and then loaded onto a Waters Symmetry C18 column (7.5 cm × 4.6 mm, 3.5 μm particle size). The column temperature was maintained at 30°C for PAs. The mobile phase was methanol-H_2_O (59:41, v:v). The PA peaks were detected with a UV detector at 230 nm at a flow rate of 0.7 ml min^-1^.

### Total RNA Extraction and Gene Expression Analysis

Frozen leaves (0.5 g) were ground in liquid nitrogen with 2% polyvinylpyrrolidone and extracted with an RNA purification reagent (Invitrogen Inc., Carlsbad, CA, USA) according to the manufacturer’s instructions. The RNA content was determined with an ultraviolet spectrophotometer (Cary 50, Varian, Agilent), and the integrity of the RNA was evaluated by 1% agarose gel electrophoresis. Total RNA was reverse-transcribed into first-strand cDNAs using M-MLV reverse transcriptase (TaKaRa, Dalian, China). A 10-μl real-time PCR reaction contained the following: 0.6 μl of forward and reverse primers for the sugar biosynthesis-related genes, PA biosynthesis-related genes, betaine aldehyde dehydrogenase (*SoBADH*), choline monooxygenase (*SoCMO*), and aquaporin (*SoPIP1;2*) (listed in Supplementary Table S1); 1 μl of cDNA (equivalent to 10 ng of mRNA) and 5 μl of FastStart Universal SYBR Green Master Mix (ROX, Mannheim, Germany). The amplification and detection of the dsDNA synthesis of these genes were performed using the PCR conditions described in Supplementary Table S1. Three independent replicates were performed for each sample. The comparative threshold cycle (C_t_) method was used to determine the relative amount of gene expression. The glyceraldehyde-3-phosphate dehydrogenase gene (*SoGADPH*) was used as an internal control. The mRNA transcriptional abundance value of the genes was expressed as 2^-ΔΔCt^ (Livak and Schmittgen, 2001). A LightCycler 480II Real-Time PCR System (Roche, Bern, Switzerland) was used to perform qRT-PCR.

### Statistical Analysis

At least six leaves were used to measure gas exchange and chlorophyll fluorescence quenching. At least three replicates were conducted for physiological and biochemical analyses. One-way or two-way ANOVA procedure with the SPSS 19.0 software (SPSS Inc., Chicago, IL, USA) were used for analyzing statistical significances, and the results are expressed as the mean values ± SE. *Post hoc* comparisons were tested using the Tukey test at a significance level of *P*< 0.05.

## Results

### Effect of H_2_S Donor on Plant Water Stress Responses

**FIGURE 1:**
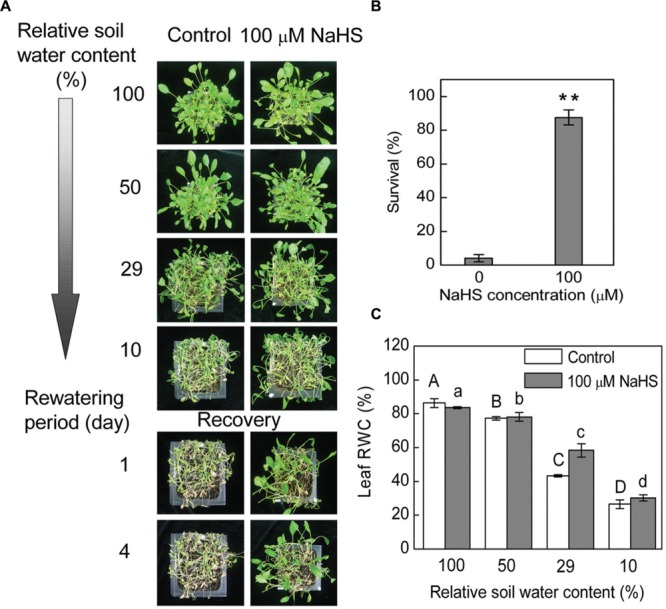
**Plant water stress response was analyzed in 6-week-old NaHS-treated *Spinacia oleracea* seedlings grown under drought and rewatering conditions.** Four containers of each treatments were determined in three independent experiments. Relative soil water content is the soil water relative to the soil water at day 0 of withholding water and is the average of four containers. The photograph in **(A)** illustrates a representative result of one replicate from one experiment. Four days after rewatering, plant survival was determined (mean ± SE, *n* = 4) **(B)**. In a separate experiment, 6-week-old seedlings were exposed to water deficit stress by withholding water. Leaf RWC (mean ± SE, *n* = 6) was determined **(C)**. In **(B)**, the significant level of the difference between control and treatment is indicated by two asterisks **P< 0.01. In **(C)**, the columns labeled with different letters indicate significant differences at *P* < 0.05.

Under drought conditions, and thus under low relative SWC, *S. oleracea* seedlings treated with 100 μM NaHS, a donor of H_2_S, survived better than untreated control plants (**Figure 1A**). Most of the control plants wilted at 10 ± 1.5% SWC, while most of the NaHS-treated plants presented less severe leaf wilting symptoms (**Figure 1A**). Additionally, on the first day after re-watering, the NaHS-treated seedlings recover more quickly than the control plants (**Figure 1A**). Moreover, more than 80% of the NaHS-treated plants and <10% of the control plants survived to the fourth day after re-watering (**Figure 1B**). Increased drought survival of the NaHS-treated plants was associated with a higher capacity to maintain RWC than the control plants at 29 ± 2.5% SWC (**Figure 1C**). To understand the physiological mechanism through which the NaHS-treated plants acquired water stress tolerance by maintaining higher leaf water content under low soil moisture conditions, we analyzed the effect of different concentrations of NaHS on the RWC of detached leaves of *S. oleracea* seedlings. The RWC of detached *S. oleracea* seedling leaves treated with various concentrations of NaHS (10–1000 μM) were significantly higher than that of control plants, with an optimal NaHS concentration of 100 μM (Supplementary Figure S1A). Indeed, the detached leaves treated with 100 μM NaHS exhibited the highest leaf RWC and the lowest leaf water loss ratio of all plants (Supplementary Figures S1B,C). The leaf water loss of NaHS-treated plants decreased by ∼10% compared with that of the control plants (Supplementary Figure S1D).

### Effect of H_2_S Donor on the Endogenous H_2_S Concentration

**FIGURE 2:**
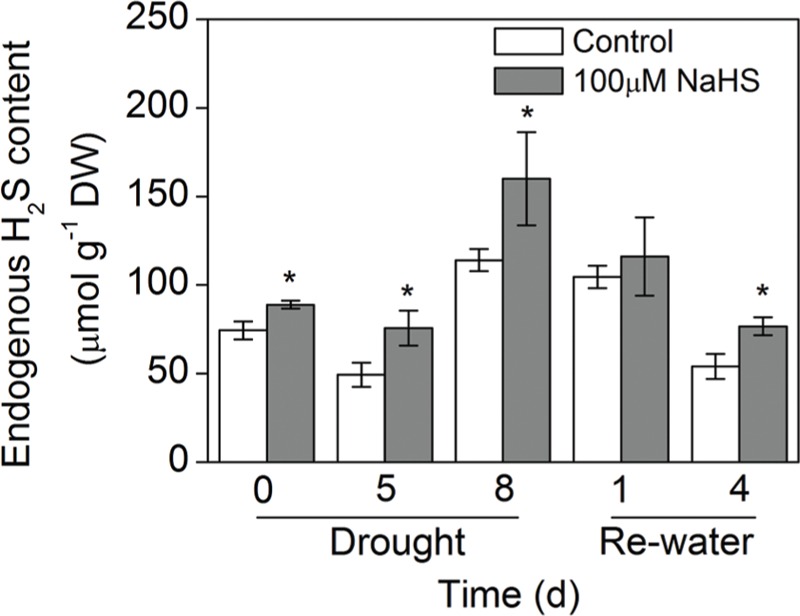
**Effect of H_2_S donor NaHS on the endogenous H_2_S concentration in 6-week-old *S. oleracea* seedlings leaves after drought for 0, 5, and 8 days and rewatering for 1 and 4 days.** The significant level of the difference between control and treatment is indicated by an asterisk ^∗^*P*< 0.05.

To determine whether exogenous H_2_S affects endogenous H_2_S concentrations in *S. oleracea* seedlings suffering from drought stress, we measured the endogenous H_2_S concentration of *S. oleracea* seedling leaves. As shown in **Figure 2**, 8 days of drought stress promoted endogenous H_2_S production in *S. oleracea* seedlings. Moreover, exogenously applied NaHS also increased the production of endogenous H_2_S under drought stress. However, re-watering for 4 days inhibited the drought-induced endogenous production of H_2_S in *S. oleracea* seedling leaves (**Figure 2**).

### Effect of the H_2_S Donor on Leaf Photosynthesis and Electron Transport Efficiency of PSII

**FIGURE 3:**
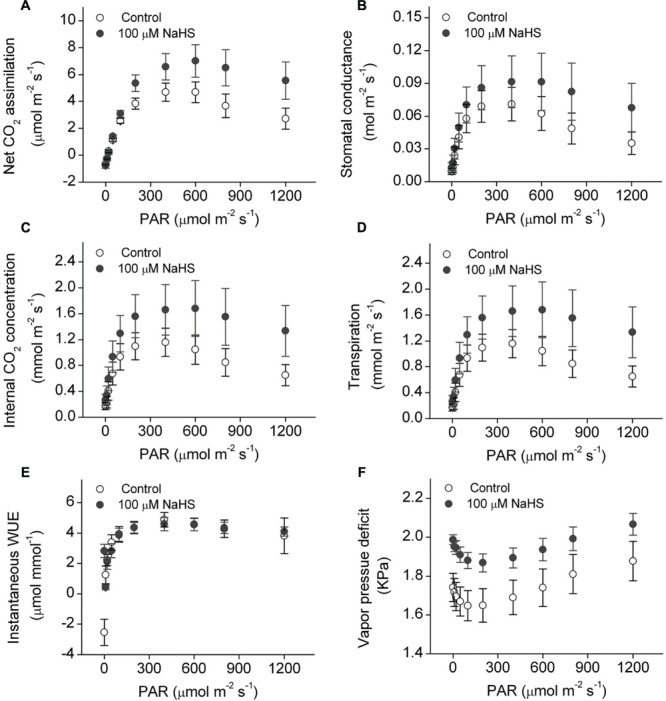
**Effect of H_2_S donor NaHS on net CO_2_ assimilation **(A)**, stomatal conductance **(B)**, internal CO_2_ concentration **(C)**, transpiration **(D)**, instantaneous WUE **(E)**, and vapor pressure deficit **(F)** of 6-week-old *S. oleracea* seedlings leaves under drought condition.** Each value represents the mean ± SE (*n* = 6).

There is a close relationship between water stress and photosynthesis in plants. Accordingly, we measured the photosynthesis of *S. oleracea* seedlings treated with NaHS under drought condition. The net CO_2_ assimilation rate of the NaHS-treated plants was significantly increased compared with that of control plants (**Figure 3A**). Additionally, the stomatal conductance of the NaHS-treated plants was somewhat higher than that of the control plants, although the difference was not statistically significant (**Figure 3B**). The internal CO_2_ concentration in the leaves of NaHS-treated plants was higher than in those of the control plants (**Figure 3C**). Additionally, at saturating light levels, the leaf transpiration rate of the NaHS-treated plants was higher than that of the control plants (**Figure 3D**). However, the instantaneous WUE values of the NaHS-treated plants and the control plants were similar (**Figure 3E**). Additionally, the VPD of the NaHS-treated plants was higher than that of the control plants (**Figure 3F**). Moreover, the *R*d, *L*cp, *L*sp, *AQE*, and *P*max of *S. oleracea* plants were calculated by modeling the response of the leaf Pn to PAR via a non-rectangular hyperbola (**Table 1**). *L*cp, *L*sp, and *P*max were notably higher in the NaHS-treated plants than in the controls. These results demonstrate that NaHS treatment increased the photosynthesis of *S. oleracea* seedlings under drought condition.

**Table 1:** Effects of H_2_S on apparent quantum yield (*AQE*), dark respiration (*R*d), low light compensation point (*L*cp), high light saturation point (*L*sp), and maximum net photosynthetic rate (*P*max) of *S. oleracea* seedlings under drought condition.

Variables	Control	100 μM NaHS
*AQE*	0.050 ± 0.005	0.051 ± 0.003
*R*d (μmol CO_2_ m^-2^ s^-1^)	0.814 ± 0.096	0.890 ± 0.093
*L*cp (μmol m^-2^ s^-1^)	16.11 ± 1.00	19.30 ± 0.80^∗^
*L*sp (μmol m^-2^ s^-1^)	236.9 ± 39.85	382.9 ± 24.01^∗∗^
*P*max (μmol CO_2_ m^-2^ s^-1^)	4.92 ± 0.71	7.64 ± 1.01^∗^

*Data are presented as mean ± SE. The significant level of difference between control and treatment is indicated by asterisk ^∗^*P*< 0.05 and ^∗∗^*P*< 0.01.*

**FIGURE 4:**
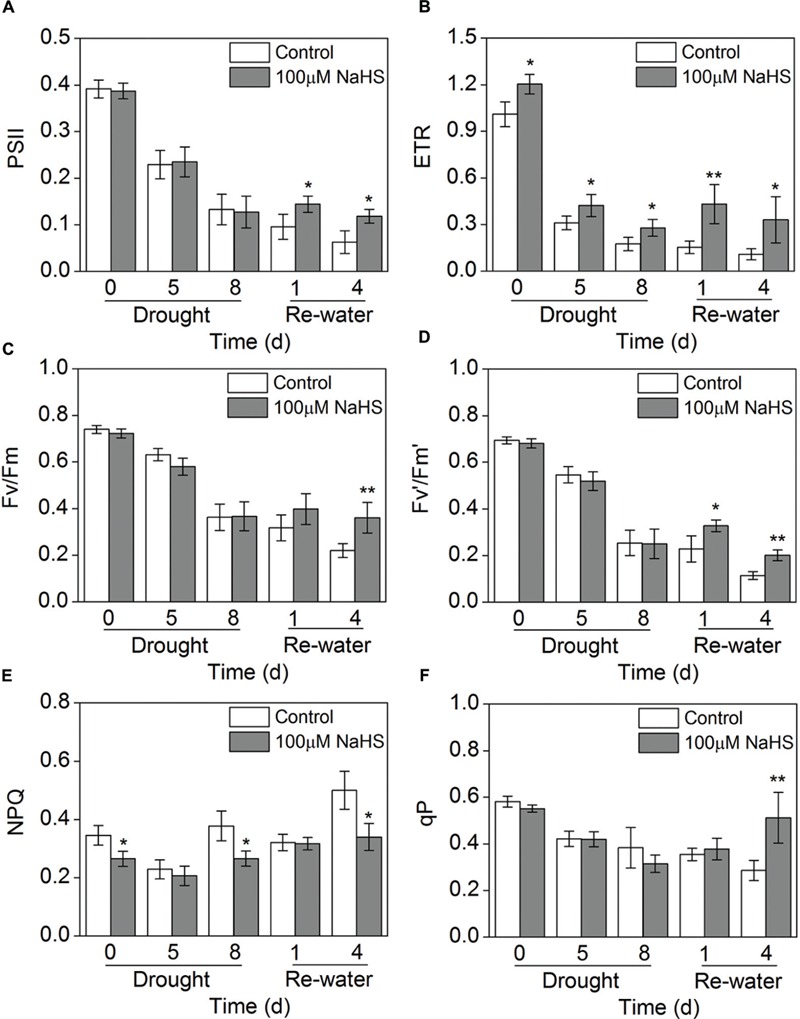
**Effect of H_2_S donor NaHS on PSII **(A)**, ETR **(B)**, Fv/Fm **(C)**, Fv′/Fm′**(D)**, NPQ **(E)** and qP **(F)** in 6-week-old *S. oleracea* seedlings leaves after drought for 0, 5, and 8 days and rewatering for 1 and 4 days.** The significant level of the difference between control and treatment is indicated by an asterisk ^∗^*P*< 0.05 and ^∗∗^*P*< 0.01.

Chlorophyll fluorescence is considered a tool for interpreting stress tolerance in plants. Therefore, we measured this key trait in *S. oleracea* seedlings under drought stress. Parameters indicative of chlorophyll fluorescence (PSII, ETR, Fv/Fm, Fv′/Fm′ and qP) were significantly decreased in the NaHS-treated and the control plants when the seedlings were under drought condition for 5 and 8 days compared with the water sufficient plants (0 day) (**Figures 4A–D,F**). Interestingly, PSII and Fv′/Fm′ in the NaHS-treated plants were higher than in the control plants on the first and fourth day after re-watering (**Figures 4A,D**). The ETR of the NaHS-treated plants was obviously increased compared with that of control plant throughout the entire treatment period, including 8 days of drought and 4 days of re-watering (**Figure 4B**). In contrast, NPQ in the NaHS-treated plants was significantly decreased compared with that of control plants throughout the entire treatment period (**Figure 4E**). The above results show that compared with the control plants, the NaHS-treated *S. oleracea* plants exhibited the high electron transport efficiency under drought condition.

### Effect of H_2_S Donor on Leaf Water Potential and Osmotic Potential

**FIGURE 5:**
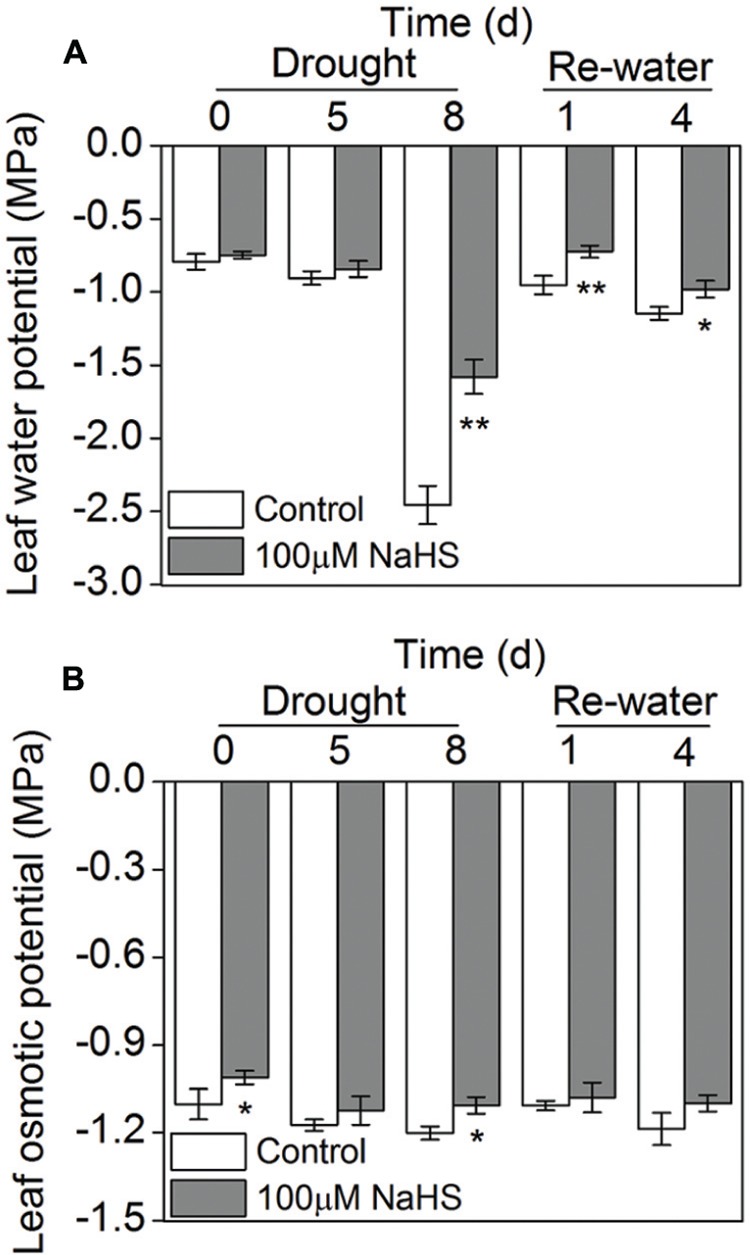
**Effect of H_2_S donor NaHS on leaf water potential **(A)** and leaf osmotic potential **(B)** in 6-week-old *S. oleracea* seedlings leaves after drought for 0, 5, and 8 days and rewatering for 1 and 4 days.** The significant level of the difference between control and treatment is indicated by an asterisk ^∗^*P*< 0.05 and ^∗∗^*P*< 0.01.

The leaf water potential of the NaHS-treated plants and the control plants grown under drought condition underwent a significant decrease, especially on the eighth day after drought, when the NaHS-treated plant exhibited a higher leaf water potential than the control plants. After re-watering, the leaf water potential experienced a sharp increase in the NaHS-treated and control plants, and the NaHS-treated plants again showed a higher leaf water potential than the control plants (**Figure 5A**). However, the leaf osmotic potential of the NaHS-treated plants and the control plants showed no significant difference under drought and re-watering conditions except for the drought at 0 and 8 days (**Figure 5B**).

### Effect of H_2_S Donor on Superoxide Radical Production, Hydrogen Peroxide Production, and Lipid Peroxidation Content

**FIGURE 6:**
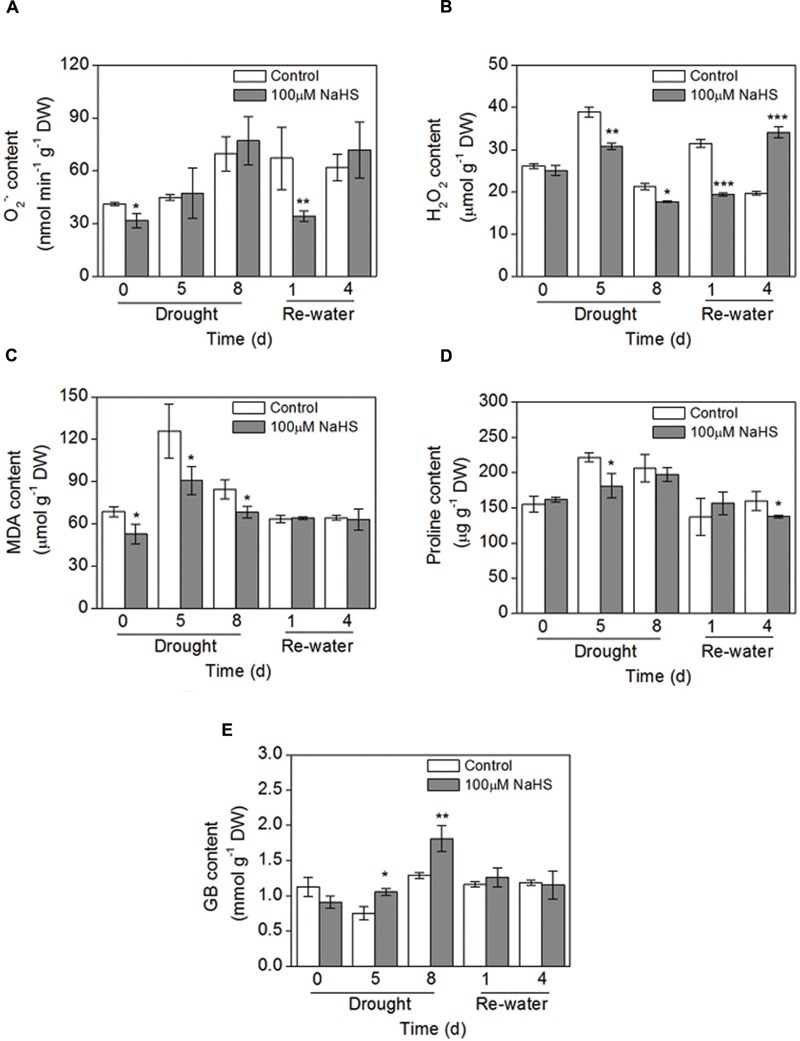
**Effect of H_2_S donor NaHS on the production rate of $O_{2}^{–•}$**(A)**, H_2_O_2_ content **(B)**, MDA content **(C)**, proline content **(D)** and glycinebetaine (GB) content **(E)** in 6-week-old *S. oleracea* seedlings leaves after drought for 0, 5, and 8 days and rewatering for 1 and 4 days.** The significant level of the difference between control and treatment is indicated by an asterisk ^∗^*P*< 0.05, ^∗∗^*P*< 0.01, and ^∗∗∗^*P*< 0.001.

To further confirm the protective role of H_2_S in the plant response to drought stress, some simple physiological parameters were measured in *S. oleracea* seedlings. The production rate of superoxide radical ($O_{2}^{–•}$) had no obvious difference between the NaHS-treated plants and the control plants under drought condition. However, on the first day after re-watering, the $O_{2}^{–•}$ content significantly decreased in the NaHS-treated plants (**Figure 6A**). Additionally, there was a significant decrease in the accumulation of H_2_O_2_ in NaHS-treated plants compared with the control plants throughout the treatment period, except for 4 days after re-watering (**Figure 6B**). Similarly, under drought condition, the content of lipid peroxidation (MDA) underwent a sharp decrease in the NaHS-treated plants compared with the control plants, but the MDA content was not significantly different between treatments after re-watering (**Figure 6C**). These results show that H_2_S can protect *S. oleracea* seedlings against drought through the inhibition of oxidative stress.

### Effect of H_2_S Donor on Proline and GB Content

NaHS treatment had no obvious effect on the proline content under drought condition, but on the fourth day after re-watering the proline content had an obvious decrease in the NaHS-treated plants (**Figure 6D**). Furthermore, GB content in NaHS-treated plants had obvious increase under drought stress for 5 and 8 days compared with the control plants, respectively (**Figure 6E**). However, NaHS had no significant effect on the GB content under re-watering for 1 and 4 days compared with the control plants, respectively (**Figure 6E**). Besides, we also found that drought stress for 8 days could increase the GB content of *S. oleracea* seedlings leaves compared with the control plants (0 day) (**Figure 6E**).

### Effect of H_2_S Donor on Sugar Biosynthesis

**FIGURE 7:**
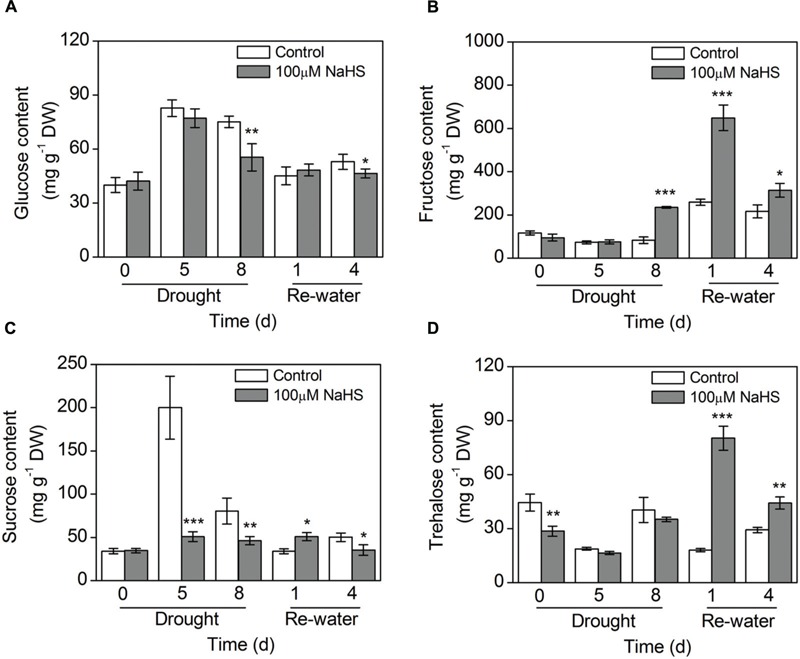
**Effect of H_2_S donor NaHS on glucose content **(A)**, fructose content **(B)**, sucrose content **(C)**, and trehalose content **(D)** of 6-week-old *S. oleracea* seedlings leaves after drought for 0, 5, and 8 d, and rewatering for 1 and 4 days.** The significant level of the difference between control and treatment is indicated by an asterisk ^∗^*P*< 0.05, ^∗∗^*P*< 0.01, and ^∗∗∗^*P*< 0.001.

The glucose content in the NaHS-treated plants decreased to varying degrees compared with the control plants under drought and re-watering conditions (**Figure 7A**). In contrast, the fructose content in the NaHS-treated plants increased considerably compared with that of control plants under the same drought and re-watering conditions (**Figure 7B**). The sucrose content in the NaHS-treated plants decreased significantly compared with that of control plants under drought condition, but the content of this sugar increased notably on the first day of re-watering (**Figure 7C**). Similarly, the trehalose content in the NaHS-treated plants experienced a significant decrease compared with the control plants under drought condition, but after re-watering the trehalose content sharply increased in the NaHS-treated plants (**Figure 7D**).

### Effect of H_2_S Donor on Polyamine Biosynthesis

**FIGURE 8:**
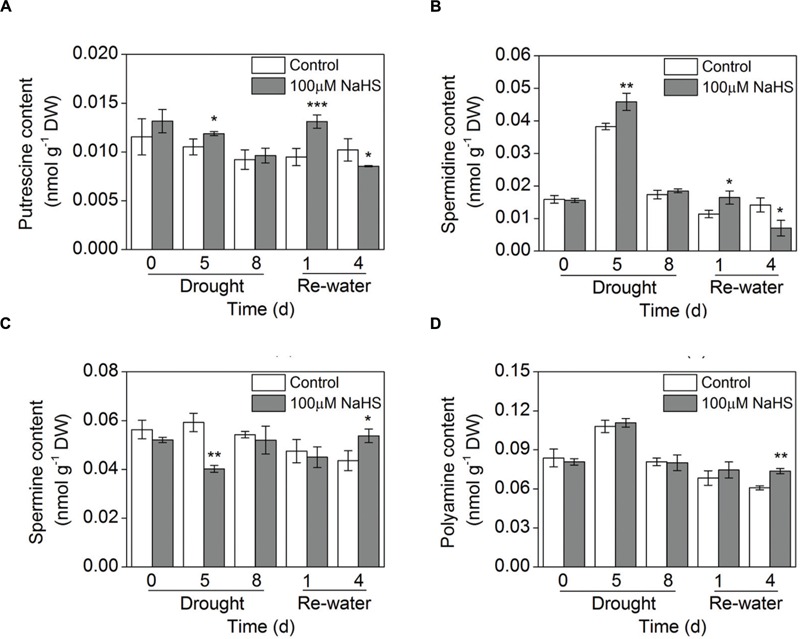
**Effect of H_2_S donor NaHS on free polyamine including putrescine content **(A)**, spermidine content **(B)**, spermine content **(C)**, and the total polyamine content **(D)** of 6-week-old *S. oleracea* seedlings leaves after drought for 0, 5, and 8 d and rewatering for 1 and 4 days.** The significant level of the difference between control and treatment is indicated by an asterisk ^∗^*P*< 0.05, ^∗∗^*P*< 0.01, and ^∗∗∗^*P*< 0.001.

The concentration of total free and conjugated polyamines (PAs) was increased in leaves of the NaHS-treated plants compared with that of control plants under similar drought and re-watering conditions (**Figure 8D**; Supplementary Figure S2D). In particular, this difference was most evident on the fourth day of re-watering (**Figure 8D**). Moreover, the free Put content was significantly increased in the NaHS-treated plants after 5 days of drought and 1 day of re-watering (**Figure 8A**). Similarly, the free Spd content increased substantially in the NaHS-treated plants after 5 days of drought and 1 day of re-watering (**Figure 8B**). In contrast, NaHS treatment significantly decreased the free Spm content after 5 days of drought, but on the fourth day of re-watering, the Spm content increased notably (**Figure 8C**). Moreover, the conjugated Put content was significantly increased in the NaHS-treated plants after 1 and 4 days of re-watering (Supplementary Figure S2A). Similarly, the conjugated Spd content increased notably in the NaHS-treated plants under drought and re-watering conditions (Supplementary Figure S2B). Additionally, NaHS treatment altered the conjugated Spm content under drought and re-watering conditions (Supplementary Figure S2C).

### Effect of H_2_S Donor on Transcriptional Expression of Sugar Biosynthesis-Related Genes

**FIGURE 9:**
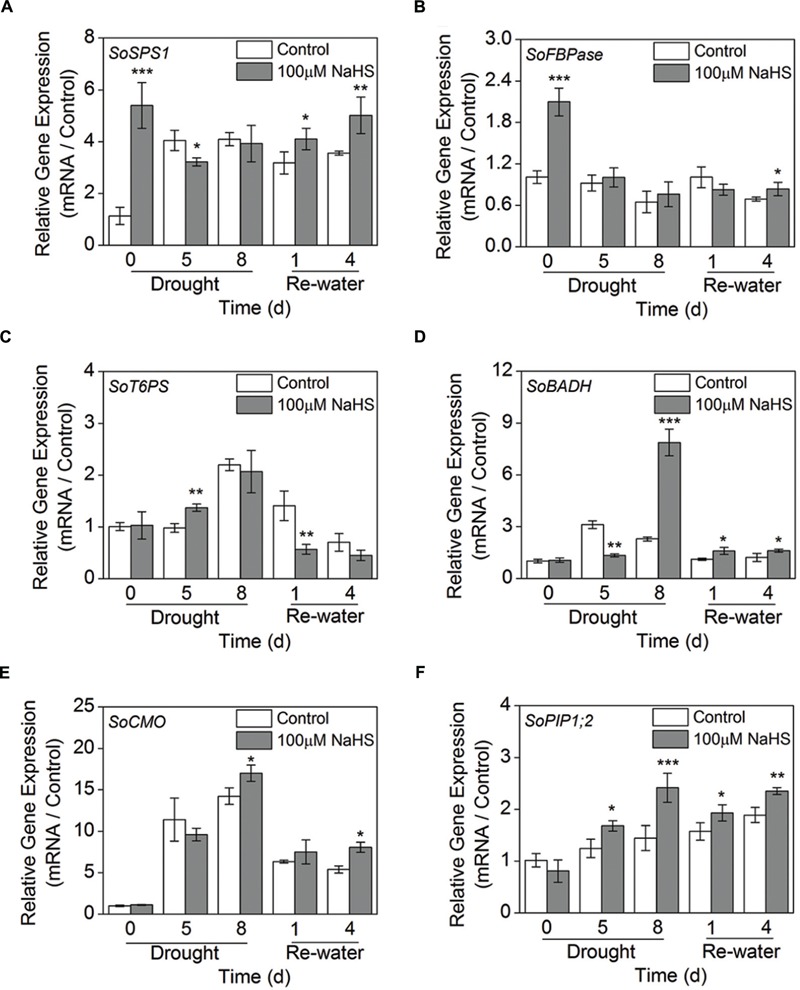
**Effect of H_2_S donor NaHS on the relative transcript abundance of *SoSPS1***(A)**, *SoFBPase***(B)**, *SoT6PS***(C)**, *SoBADH***(D)**, *SoCMO***(E)**, and *SoPIP1;2***(F)** mRNA accumulation in the leaves of *S. oleracea* seedlings after drought for 0, 5, and 8 days and rewatering for 1 and 4 days.** Transcriptional expression of the six genes was measured using real-time qPCR and were normalized with a reference gene (*SoGADPH*). Mean values ± SE were calculated from three independent experiments. The significant level of the difference between control and treatment is indicated by an asterisk ^∗^*P*< 0.05, ^∗∗^*P*< 0.01, and ^∗∗∗^*P*< 0.001.

To further study the molecular mechanism of H_2_S-alleviated drought stress in plants, we examined the expression levels of the sugar biosynthesis-related genes, GB biosynthesis-related genes such as *SoBADH, SoCMO*, and *SoPIP1;2* using RT-qPCR analysis in *S. oleracea* seedlings. The expression level of sucrose phosphate synthase (*SoSPS1*) in the NaHS-treated plants was significantly increased compared to that of control plants after 0 day of drought and 1 and 4 days of re-watering (**Figure 9A**). Additionally, the expression level of fructose-1,6-bisphosphatase (*SoFBPase*) increased to varying degrees in the NaHS-treated plants (**Figure 9B**). Moreover, the expression level of trehalose-6-phosphate synthase (*SoT6PS*) increased clearly in the NaHS-treated plants after 5 days of drought, but significantly decreased after 1 day of re-watering (**Figure 9C**). Furthermore, the expression level of *SoBADH* in the NaHS-treated plants increased significantly compared with that of control plants after 8 days of drought and 1 and 4 day of re-watering, but the expression level of *SoBADH* decreased sharply in the NaHS-treated plants after 5 days of drought (**Figure 9D**). Additionally, the expression abundance of *SoCMO* in the NaHS-treated plants was evidently enhanced compared with that in control plants after 8 days of drought and 4 days of re-watering, but no significant difference were detected during other treatment periods (**Figure 9E**). Interestingly, the expression level of *SoPIP1;2* in the NaHS-treated plants was significantly increased compared with that in the control plants after 5 and 8 days of drought, and an analogous result was also observed after 1 and 4 days of re-watering (**Figure 9F**).

### Effect of H_2_S Donor on Transcriptional Expression of Polyamines Biosynthesis-Related Genes

**FIGURE 10:**
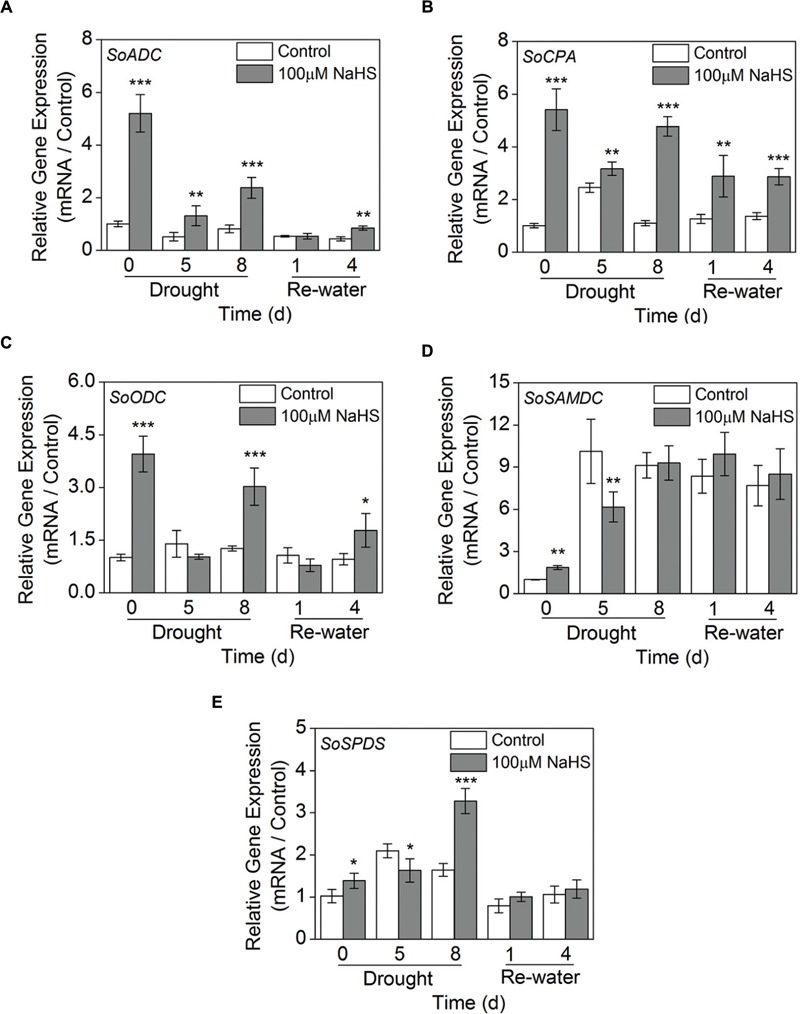
**Effect of H_2_S donor NaHS on the relative transcript abundance of *SoADC***(A)**, *SoCPA***(B)**, *SoODC***(C)**, *SoSAMDC***(D)**, and *SoSPDS***(E)** mRNA accumulation in the leaves of *S. oleracea* seedlings after drought for 0, 5, and 8 days and rewatering for 1 and 4 days.** Transcriptional expression of the six genes was measured using real-time qPCR and were normalized with a reference gene (*SoGADPH*). Mean values ± SE were calculated from three independent experiments. The significant level of the difference between control and treatment is indicated by an asterisk ^∗^*P*< 0.05, ^∗∗^*P*< 0.01, and ^∗∗∗^*P*< 0.001.

We examined the expression levels of 5 genes involved in PA biosynthesis in *S. oleracea* leaves: arginine decarboxylase (*SoADC*), *N*-carbamoylputrescine amidohydrolase (*SoCPA*), ornithine decarboxylase (*SoODC*), *S*-adenosyl-Met-decarboxylase (*SoSAMD*), and spermidine synthase (*SoSPDS*) using RT-qPCR analysis. The expression level of *SoADC* significantly increased in the NaHS-treated plants compared with the control plants after 0, 5, and 8 days of drought, and a comparable result was also observed after 4 days of re-watering (**Figure 10A**). Moreover, NaHS treatment significantly increased the expression level of *SoCPA* throughout the experiment in *S. oleracea* seedlings (**Figure 10B**). Similarly, NaHS treatment also clearly increased the expression level of *SoODC* after 0 and 8 days of drought and 4 days of re-watering in *S. oleracea* seedlings (**Figure 10C**). On the contrary, the expression level of *SoSAMD* had a significant decrease in the NaHS-treated plants after 0 and 5 days of drought, but no significant difference was observed at other treatment periods (**Figure 10D**). Furthermore, the expression level of *SoSPDS* in the NaHS-treated *S. oleracea* seedlings increased to varying degrees compared with that of the control plants, particularly after 8 days of drought (**Figure 10E**).

## Discussion

### H_2_S Attenuates Drought Stress-Induced Water Scarcity

Plants have evolved a variety of strategies to resist or avoid drought stress and reduce the damage caused by lack of water. These strategies include regulating stomatal closure and reducing transpiration, inhibiting the growth rates of leaves or stems, maintaining or increasing the hydraulic conductance of roots and shoots, synthesizing osmotic solutes to maintain cell turgor, and producing antioxidant proteins to retard chlorophyll decomposition (Wilkinson and Davies, 2010). In the present study, under drought stress condition, the survival ratio of plants, the leaf RWC, and photosynthesis were higher in the NaHS-treated plants than in the control plants (**Figures 1** and **3**, Supplementary Figure S1). In most plants, the essential goals of drought resistance strategies are to increase water uptake from roots or reduce water loss from the leaves (Yin et al., 2013). In the present study, NaHS-treated plants exhibited significantly less water loss than the control plants (Supplementary Figures S1C,D). Moreover, although the transpiration rate and stomatal conductance were higher in the NaHS-treated plants than in the control plants, the NaHS-treated plants were able to maintain a higher leaf RWC than the control plants, indicating that the alleviation of water stress was not due to the regulation of stomatal closure in this study (**Figures 1C** and **3B,D**). In this study, under drought stress, the NaHS-treated plants exhibited a high photosynthetic rate compared with the control plants. Under drought stress, the H_2_S concentration was found that to be higher in NaHS-treated plants than in the untreated plants (**Figure 2**). These results show that NaHS application enhanced *S. oleracea* seedling resistance to drought stress. Thus, our results indicate that H_2_S controlled plant the RWC of plant leaves under drought stress. Comparable results have also been observed with silicon, but although silicon alleviated drought stress, the RWC and stomatal conductance were higher in silicon-treated plants than in the untreated plants (Yin et al., 2013). Importantly, in contrast to the above finding, Jin et al. (2013) reported that H_2_S plays a vital role in the regulation of stomatal closure in the plant response to drought stress. Some possible explanations for this discrepancy include the identity of the plant species, the stress applied, the treatment time and the specific environmental conditions used. The above results showed that H_2_S can attenuate drought stress in *S. oleracea* seedlings. However, the precise mechanism by which H_2_S attenuates drought stress in *S. oleracea* seedlings remains unclear.

### H_2_S Enhances the Tolerance of Plants to Drought Stress by Increasing Osmoprotectants Content

Under stress condition, plants that have evolved in high-temperature, saline or drought environments usually accumulate three types of osmoprotectants: non-structural sugars and polyols, betaines and allied compounds, and amino acids such as proline. A previous study indicated that salinity decreased cell expansion in tomato plants, which was related to decreases in osmotic and water potentials (Romero-Aranda et al., 2001). These results are consistent with those observed in our experiments (**Figures 5A,B**). The specific composition of osmoprotectants in plant leaves under drought conditions could be implicated in the specific response observed here. In the present study, the accumulation of soluble sugars, including glucose and sucrose, in the NaHS-treated *S. oleracea* leaves was lower than in the control plants, but the fructose and trehalose contents were significantly increased compared with those of the control plants, suggesting that NaHS enhances drought resistance by regulating the accumulation of soluble sugars, especially the contents of fructose and trehalose (**Figure 7**). However, under drought and re-watering condition, the expression levels of *SoSPS1* and *SoFBPase* in the NaHS-treated plants increased to varying degrees compared with those in the control plants (**Figures 9A,B**). Similarly, Rivero et al. (2014) reported that salt and heat treatment specifically up-regulated the expression levels of the *FBPase* gene. Moreover, the function of this enzyme in the pathway of gluconeogenesis may be a limiting step in the synthesis of starch and trehalose because the activity of FBPase is very important for glucose regeneration, which is the main carbon skeleton in trehalose and starch (Rivero et al., 2014).

Additionally, the function of trehalose in stress response remains controversial, though it has been shown stabilize membranes and protect proteins in desiccated tissues and has been suggested to function as a chemical chaperone (Rivero et al., 2014). However, trehalose levels in nature and even in engineered plants usually remain well below 1 mg g^-1^ FW, suggesting that trehalose does not act as a compatible solute, but has an alternative function (Garg et al., 2002). The molecular relevance and roles that have been proposed and partially demonstrated for trehalose in plants have been reviewed by Ponnu et al. (2011). Additionally, trehalose-6-phosphate (T6P), a precursor of trehalose, has been proven to play a key role in regulating carbohydrate metabolism by inducing starch synthesis in the plastid. Furthermore, trehalose has also been linked to, plant development, cell growth and the induction of photosynthesis capacity. Previous studies have shown that a decrease in the T6P concentration results in the induction of genes involved in photosynthesis-related processes, so that more carbon is used for the growing cell, to supplement the lack of carbon (Ponnu et al., 2011). In the present study, the trehalose content in the NaHS-treated plants significantly increased after 1 and 4 days of re-watering (**Figure 7D**). Moreover, the NaHS-treated plants exhibited obvious increases in photosynthesis and PSII efficiency compared with the control plants. Our results show that the increased in photosynthesis capacity and PSII efficiency may also be explained by the regulatory role of trehalose during this process. Furthermore, Garg et al. (2002) suggested that a modulated carbohydrate metabolism could explain the lowered photo-oxidative damage associated with the high trehalose levels, which is consistent with our findings.

It is well established that various abiotic stresses such as salinity, drought, cold, and high-temperatures can lead to the accumulation of ROS. Osmoprotective compounds can directly remove ROS or cause the protection of antioxidant enzymes. In this regard, proline has been found to be a singlet oxygen quencher during osmotic stress because it can reduce the damage of ROS in different plants. As an example, the soaking of buds in proline and GB solutions greatly reduces the production of H_2_O_2_, improves the accumulation of soluble sugar and protects the developing tissues from the effects of stress. Similarly, our results also showed that the NaHS-treated plants could accumulate high GB contents and the up-regulated expression of the *SoBADH* and *SoCMO* genes under drought conditions (**Figures 6E** and **9D,E**), which result in lower H_2_O_2_ accumulation and MDA contents in *S. oleracea* leaves (**Figures 6B,C**). However, NaHS treatment had no significant effect on the content of proline in *S. oleracea* seedlings (**Figure 6D**). A possible explanation for this discrepancy is that proline accumulation results from the activation of its synthesis and an inhibition of its degradation at both the transcriptional and post-transcriptional levels. Therefore, our results indicate that NaHS plays an important role in protecting plants from drought stress by influencing the accumulation of osmoprotective compounds and related gene expression.

### H_2_S Enhances the Tolerance of Plants to Drought Stress by Increasing Polyamine Biosynthesis

Changes in PA levels and the accumulation of osmoprotective compounds are a common plant response to the lack of water and are associated with plant drought tolerance (Yin et al., 2014). The role of PAs in enhancing drought tolerance has been determined such as increasing the ability of antioxidants, taking effect as stress-signaling regulators, inducing stomatal closure and enhancing leaf water content (Shi et al., 2010; Yin et al., 2014; Muilu-Makela et al., 2015). In addition, the activation of ADC and/or ODC may also stimulate PA biosynthesis in plant tissues because these two enzymes catalyzed the production of Put. Generally, under stress conditions, the pathway of ADC is easily activated; in contrast, ODC is essential for cell proliferation and the biosynthesis of root Put (Tiburcio et al., 2014). Additionally, in this study, the application of NaHS under drought condition resulted in the increased biosynthesis of free and conjugated PAs biosynthesis (**Figure 8**; Supplementary Figure S2). Moreover, to further study how H_2_S regulates PA biosynthesis, the transcriptional abundances of PA biosynthesis-related genes were determined. Our results revealed that the transcription level of *SoADC* was up-regulated by NaHS under drought condition (**Figure 10A**). Many previous studies have reported that the ADC pathway is closely related to drought stress and that ADC can regulate the biosynthesis of Put (Yin et al., 2014; Lulai et al., 2015). In fact, the two enzymes agmatine iminohydrolase (AIH) and CPA are also involved in Put biosynthesis in the ADC pathway. Here, the expression level of *SoCPA* was up-regulated by NaHS (**Figure 10B**). Interestingly, the transcription levels of *SoODC* were significantly increased in the NaHS-treated plants grown under drought condition (**Figure 10C**). Accordingly, these results show that H_2_S mediated Put production through both the ADC and ODC pathways in *S. oleracea* seedlings. Additionally, SAMDC is a rate-limiting enzyme in Spd and Spm biosynthesis, because SAMDC has a short half-life. The transcriptional level of *SAMDC* is induced under different abiotic stresses, and the overexpression of *SAMDC* increases PA levels and enhances stress tolerance. However, in this study, the transcription of *SoSAMDC* in the NaHS-treated plants was up-regulated under normal condition, but was down-regulated by NaHS after 5 days of drought, and there was no obvious difference between the treatment periods (**Figure 10D**). Notably, the Spd level increased markedly after 5 days of drought, whereas the Spm level decreased significantly, suggesting that *SAMDC* expression did not correlate with the levels of Spd/or Spm, probably as a result of the tight regulation of enzyme activity (**Figures 8B,D**). However, it is of note that the transcription level of *SoSPDS* in the NaHS-treated plants underwent a significant increase under drought condition (**Figure 10E**). These results may explain the changes in the Spd and Spm levels observed under drought or re-watering conditions.

Abiotic stress often enhances senescence processes, where valuable resources, accumulated during prior development are recycled for use in other growing parts of the plant. Additionally, previous studies have shown that the increase of PA contents may prevent chlorophyll loss and the inhibition of photosynthesis and thus delay leaf senescence under stress conditions (Shi et al., 2010; Glaubitz et al., 2015). In this study, under drought condition, NaHS was observed to significantly increase photosynthesis in the plants (**Figure 3A**). Meanwhile, PA contents were also increased to varying degrees, which suggests that PAs may play an important role in the maintenance of photosynthesis and plant growth and additionally may participate in H_2_S-induced drought tolerance. In contrast, if senescence leads to programmed cell death, PAs may play a vital role as a carbon and nitrogen reservoir in plants under stress condition (Moschou et al., 2012). Therefore, PA homoeostasis may be was regulated by H_2_S to protect plants from drought damage.

### Pathway of H_2_S-Induced Drought Resistance

**FIGURE 11:**
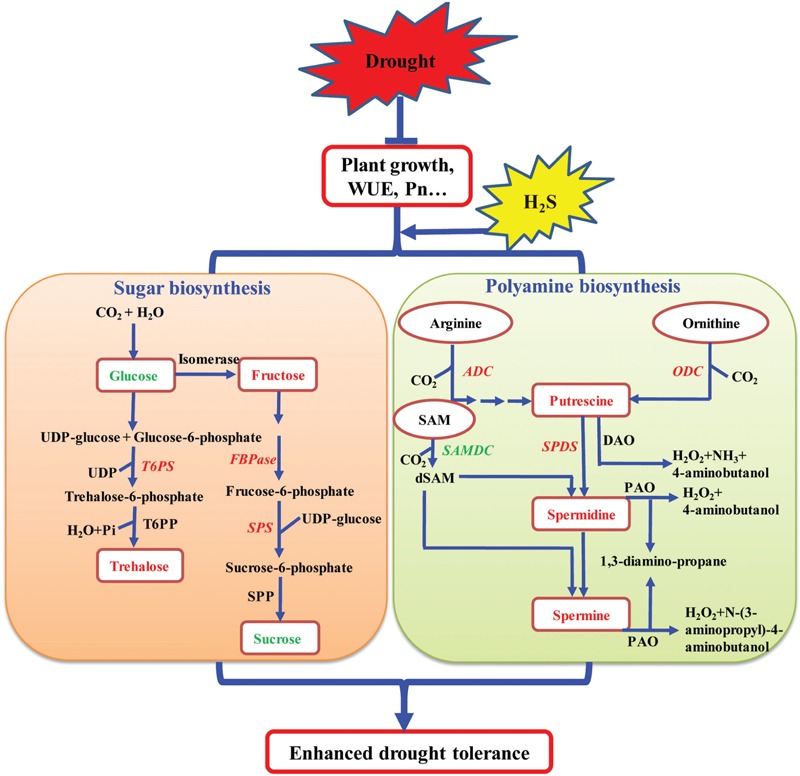
**A model depicting how H_2_S regulates the enhanced drought tolerance by mediating polyamine and sugar biosynthesis.** Under drought stress, on the one hand, the application of NaHS up-regulated the expression of polyamine biosynthetic genes encoding arginine decarboxylase (*ADC*), ornithine decarboxylase (*ODC*), and *N*-carbamoylputrescine amidohydrolase (*CPA*) (indicated by red and bold font) and down-regulated the expression of *S*- adenosyl-Met-decarboxylase (*SAMDC*) (indicated by green and bold font), resulting in elevated polyamine levels in plant tissues. On the other hand, the application of NaHS up-regulated the expression of sugar biosynthetic genes encoding trehalose-6-phosphate synthase (*T6PS*), fructose-1,6-bisphosphatase (*FBPase*), and sucrose phosphate synthase (*SPS1*) (indicated by red and bold font), resulting in the alteration of the sugar content, including elevation of the fructose and trehalose content (indicated by red and bold font) and reduction of the glucose and sucrose content (indicated by green and bold font) in plant tissues.

Based on the above results and the current knowledge concerning the mechanisms of plant response to drought stress, a signaling pathway by which H_2_S influences the biosynthesis of total PAs and sugar has been proposed. As shown in **Figure 11**, H_2_S may promote the growth and photosynthesis of plants under drought stress. Under drought stress, H_2_S up-regulates the transcription levels of *SoADC, SoCPA, SoODC*, and *SoSPDS*, leading to the increased biosynthesis and accumulation of PAs in plant tissues. Under drought stress, H_2_S may also increase the accumulation of osmoprotective compounds including soluble sugars by changing the expression levels of sugar biosynthesis-related genes. Together, high levels of PAs and soluble sugars enhance drought tolerance in plants. Our results show that H_2_S regulates drought stress response in plants by mediating certain important metabolic processes and is not limited to a mere mechanical barrier, as was previously assumed. Importantly, our study also opens new avenues of research for the growth and development of plants with enhanced tolerance to abiotic stress conditions.

## Author Contributions

JC designed the experiment and wrote the manuscript, Y-TS and X-YC conducted the experiment, W-HW and E-MH helped in data analysis and presentation, and ZS and H-LZ revised the manuscript.

## Conflict of Interest Statement

The authors declare that the research was conducted in the absence of any commercial or financial relationships that could be construed as a potential conflict of interest.
