# Supplementary material for: Hydrogen Sulfide-Mediated Polyamines and Sugar Changes Are Involved in Hydrogen Sulfide-Induced Drought Tolerance in Spinacia oleracea Seedlings
Source: Front Plant Sci. 2016 Aug 4;7:1173. doi: 10.3389/fpls.2016.01173 (PMC4972840; doi:10.3389/fpls.2016.01173)
Supplement: Supplementary file 1 [file Data_Sheet_1.PDF]

Title: Hydrogen sulfide-mediated polyamines and sugar changes are involved in hydrogen sulfide-induced drought tolerance in *Spinacia oleracea* seedlings

Authors: Juan Chen[1#], Yu-Ting Shang[1#], Wen-Hua Wang[2], Xi-Yan Chen[3], En-Ming He[2], Hai-Lei Zheng[4], and Zhouping Shangguan[1*]

FIGURE S1

The detach leaf RWC of *S. oleracea* seedlings treated with different concentrationa of NaHS (0, 10, 100, 500 and 1000 µM) for 3 h (A). The detach leaf RWC of *S. oleracea* seedlings treated with 100 µM NaHS for 0, 1, 2, 3, 4, 5, and 6 h (B). The detach leaf water loss of *S. oleracea* seedlings treated with 100 µM NaHS for 0-6 h (C). The total leaf water loss of *S. oleracea* seedlings treated with 100 µM NaHS for 0-6 h (D). Each value represents the mean ± SE (n = 30). Columns labeled with different letters indicate significant differences with P<0.05. The significant level of the difference between control and treatment is indicated by an asterisk * for P < 0.05 and ** for P < 0.01.

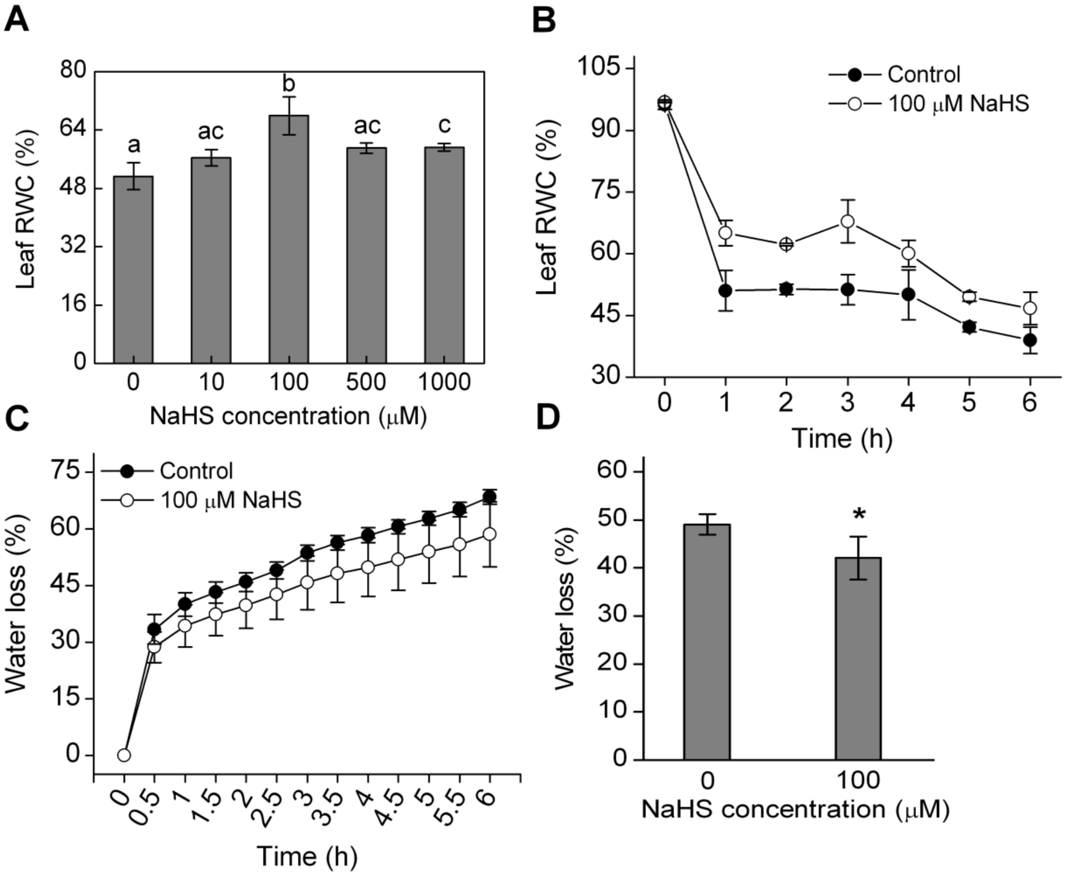

FIGURE S2

Effect of H_2S donor NaHS on conjugated polyamine including putrescine content (A), spermidine content (B), spermine content (C), and the total polyamine content (D) of 6-week-old *S. oleracea* seedlings leaves after drought for 0, 5 and 8 d and rewatering for 1 and 4 d. The significant level of the difference between control and treatment is indicated by an asterisk * for $P < 0.05$, ** for $P < 0.01$, and *** for $P < 0.001$.

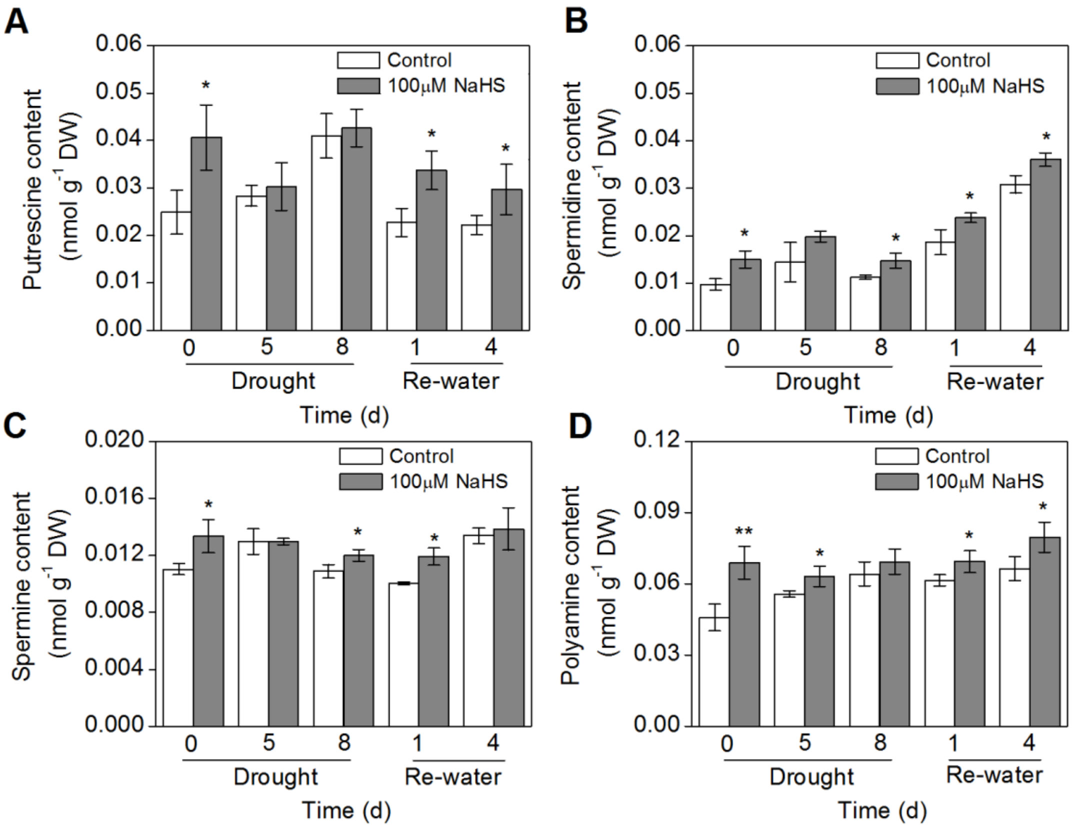

Table S1 Optimized primer sequences and reaction conditions used for real-time quantitative PCR of *SoSPS1, SoFBPase, SoT6PS, SoBADH, SoCMO, SoPIP1;2, SoADC, SoCPA, SoODC, SoSAMDC, SoSPDS* and *SoGADPH* in *S. oleracea* seedlings.

Gene	Encoded proteins	Genbank accession	Primer sequences	Optimized conditions (Tm/amplicon length)
SoSPS1	Sucrose phosphate synthase	L04803	F5'-CTAAAGGAAGAATGCGTAGAATAAG-3'<br>R5'-TCTACTACATACTTTACCTGACCACCT-3'	51 ^{o}C/175 bp
SoFBPase	Fructose-1,6-bisphosphatase	L76555	F5'-AGGTGTTTTCGAGCTGCTTGAGAT-3'<br>R5'-CGTTAGGGCTATAAATGCCAAAGAT-3'	52 ^{o}C/192 bp
SoT6PS	Trehalose-6-phosphate synthase	AF155150	F5'-ATGAGCGTTACGGTACTTCTGACTA-3'<br>R5'-GTGCTTGTGCGAGGCGATGAAT-3'	51 ^{o}C/214 bp
SoBADH	Betaine aldehyde dehydrogenase	FJ595952	F5'-GGAAACCATTGATTCTGGGAAACCT-3'<br>R5'-AACAACACCAAGGGGCTGCCTGAGA-3'	53 ^{o}C/181 bp
SoCMO	Choline monooxygenase	EF362838	F5'-CCTGAAGACGCTCATACACCTCCTA-3'<br>R5'-CTTTTATTTGATCGCTGATCCCTGC-3'	53 ^{o}C/121 bp
SoPIP1;2	Aquaporin	AY372191	F5'-CCTTCGTCCTTGTCTACACCGTCTT-3'<br>R5'-CAGTGGTCGTTCCAGTTGTTAATTTG-3'	54°C/217 bp
SoADC	Arginine decarboxylase	NC_003071	F5'-TACGAGAATCTTTATGGTGCTGCTA-3'<br>R5'-ATCACCCACTCGCATAATCCATCAA-3'	50 ^{o}C/155 bp
SoCPA	N-carbamoylputrescine amidohydrolase	NM_179770	F5'-ATTGGGTGTAGTGATACCTGTTAG-3'<br>R5'-TTAGCAAACTTCGTCTGGAAAACCT-3'	51°C/201 bp
SoODC	Ornithine decarboxylase	AF127242	F5'-CTCAAGCCGCCCGTCTCACCGTATC-3'<br>R5'-CGACATCCCGAGTTTAGCAGCTGTTTCA-3'	57°C/134 bp
SoSAMDC	S-adenosyl-Met-decarboxylase	X81414	F5'-TGTCCGTTATACCCGTGGCAGTTTC-3'<br>R5'-TGATCGTTTCAGCAGATGCAGAG-3'	53°C/191 bp

SoSPDS	Spermidine synthase	AJ251296	F5'-CATTGGAGGAGGAGATGGAGGTGTC-3'<br>R5'-TAGCCACAGATTGGAAGAAGGGTTT-3'	52°C/290 bp
SoGADPH	Glyceraldehyde-3-phosphate dehydrogenase	X15189	F5'-TGTGTGCGACATCCCCCTCGTGTCAG-3'<br>R5'-CCACCCGTTGGCTGTAACCCCACTCAT-3'	58 ^{o}C/156 bp
